# CerS2-mediated lipotoxicity and calcium signaling: a novel sequential mechanism of apoptosis in cancer

**DOI:** 10.1042/BCJ20260424

**Published:** 2026-10-15

**Authors:** Satya Narayan

**Affiliations:** Department of Physiology and Aging, College of Medicine, University of Florida, Gainesville, FL 32610, U.S.A.

**Keywords:** Apoptosis, Calcium influx, Ceramide synthase 2, DH20931 – a novel CerS2 agonist, ER stress, very-long-chain ceramides

## Abstract

The classical paradigm of the sphingolipid rheostat—balancing pro-apoptotic ceramide against pro-survival sphingosine-1-phosphate—has evolved into a complex signaling landscape where N-acyl chain length serves as the fundamental determinant of biological outcomes. This perspective explores the pivotal role of ceramide synthase 2 (CerS2) in synthesizing very-long-chain (VLC) ceramides (C22–C26) and its function as a central regulator of cellular stress responses. While CerS2 typically acts as a tumor suppressor, its activity is frequently attenuated in aggressive malignancies through alternative splicing or protein inhibition. The emergence of DH20931, a first-in-class small-molecule CerS2 agonist, is discussed as a paradigm-shifting tool for re-engaging suppressed apoptotic pathways. The present paper outlines a sequential-mechanism model for pharmacological CerS2 activation, which drives lipotoxic endoplasmic reticulum (ER) stress via the PERK-ATF4-CHOP axis and tightens the ER-mitochondria synapse through direct IP3R1 interaction. The latter mechanism facilitates lethal calcium influx, triggering the mitochondrial caspase-dependent apoptotic pathway. Furthermore, the preclinical performance of DH20931 is evaluated in models of triple-negative breast cancer (TNBC) relative to standard-of-care therapeutics. The conclusion examines the future of CerS2 as a major druggable target, emphasizing the need for companion diagnostics to identify patients most likely to benefit from agonist therapy based on specific splice variants and metabolic profiles. Combining CerS2 activation with existing chemo- or immunotherapies represents a promising synergistic strategy for treating resistant solid tumors.

## Introduction

The traditional view of cancer cell signaling has long been centered on the sphingolipid rheostat model [1–3]. This conceptual framework suggests a delicate metabolic balance between the pro-apoptotic lipid ceramide and its pro-survival counterpart, sphingosine-1-phosphate, as the primary determinant of cellular fate. However, recent advances in lipidomics and molecular biology indicate that this binary model is an oversimplification. Emerging research reveals that ceramides are not a singular entity; rather, they comprise a diverse family of lipids whose biological roles are fundamentally influenced by the length and saturation of their N-acyl chains [4–6]. This functional specificity transforms the simple metabolic switch into a sophisticated signaling interactome where the precise composition of the ceramide pool controls whether a cell survives, proliferates, or undergoes programmed death [3,7].

The architects of this diversity are the six ceramide synthase isoforms, designated CerS1, CerS2, CerS3, CerS4, CerS5, and CerS6, which are integral membrane proteins localized to the endoplasmic reticulum (ER) [8–11]. Each isoform exhibits a remarkable substrate specificity for fatty acyl-CoAs of specific chain lengths, thereby controlling the synthesis of distinct bioactive lipid messengers [12]. Among these enzymes, CerS2 is particularly notable for its ubiquitous expression and its specialized function in producing very-long-chain (VLC) ceramides, specifically those with acyl chains ranging from C22 to C26 [10,13,14]. These VLC-ceramides are not merely structural components of membranes; they are potent signaling molecules capable of orchestrating complex cellular stress responses [15,16].

In various cancers, the CerS2/VLC-ceramide axis serves as a critical relationship connecting lipid metabolism, organelle stress, and calcium (Ca^2+^) signaling. Many preclinical data suggest that CerS2 often functions as a tumor suppressor, and its down-regulation is associated with aggressive phenotypes such as breast, prostate, bladder, liver, and lung cancer [17–24]. The increased level of CerS2 has been shown to increase chemosensitivity of triple-negative breast cancer (TNBC) cells and to be associated with a better prognosis in doxorubicin-treated breast cancer patients [25]. Post-transcriptionally silenced *CERS2* expression by miR-9 contributes to tumor progression [26]. In animal studies, a poor survival of CERS2^−/−^ mice has been described [27]. These mice developed hepatocellular carcinoma at a young age of 8-10 weeks [24]. *CERS2^−/−^* mice also showed a high incidence of pheochromocytoma [28], and higher susceptibility to azoxymethane/dextran sodium sulfate-induced colitis and colon carcinoma [29]. Further, the overexpression of CerS2 has been linked with lipotoxic levels of VLC-ceramide resulting in oxidative stress and mitochondrial dysfunction [30,31]. The recent identification and characterization of DH20931, a first-in-class small-molecule agonist of CerS2, provides a significant breakthrough in the field [32,33]. These findings demonstrate that pharmacological activation of CerS2 can re-engage suppressed apoptotic pathways through a sequential-mechanism approach involving both proteotoxic stress and mitochondrial Ca^2+^ overload [32].

## Molecular architecture and catalytic specificity of CerS2

CerS2 is an essential enzyme in sphingolipid metabolism, utilizing a unique 11-residue motif to maintain specificity for C22–C26 acyl-chains [13,34]. This specificity is vital for cellular membrane integrity; its loss leads to increased membrane fluidity and the disruption of critical signaling pathways, including those governing liver homeostasis and insulin response [27]. The specific biochemical role of CerS2 in the N-acylation of sphingoid bases was first characterized by the Futerman laboratory. They established that while all six mammalian ceramide synthases catalyze the same biochemical chemical reaction, CerS2 uniquely favors very long-chain fatty acyl-CoAs (C22–C26) as the acyl donor [11,13]. CerS2 is described as a metabolic hub because it acts as a central step in two distinct cellular routes. First, *de novo* synthesis in which it acylates dihydrosphingosine (sphinganine) in the ER [14]. Second, salvage pathway, where it re-acylates sphingosine that has been recycled from the breakdown of complex sphingolipids [14]. The identification of CerS2 as the predominant source of VLC-ceramides in the liver, kidney, and brain was confirmed through the analysis of CerS2-deficient (knockout) mice. These studies showed that the loss of CerS2 leads to a depletion of ceramides with fatty acids ≥C22 in these specific tissues, confirming its essential role in maintaining their unique sphingolipid profiles [35,36].

The structural basis for the substrate specificity of CerS2 is located within its conserved catalytic TRAM-Lag1-CLN8 (TLC) domain, a hallmark of the CerS family [37,38]. Although isoforms share an overall protein architecture, chimeric studies identified key residues in an 11-residue loop between the final two putative transmembrane domains (Figure 1). When this specific 11-amino acid sequence from CerS2 is transferred into the backbone of CerS5, which normally synthesizes C16-ceramides, the resulting chimeric enzyme is reprogrammed to produce VLC-ceramides [34]. This molecular design represents a highly evolved mechanism for generating functional lipid diversity and provides a druggable hotspot for the development of allosteric modulators like DH20931 [32].

**Figure 1:**
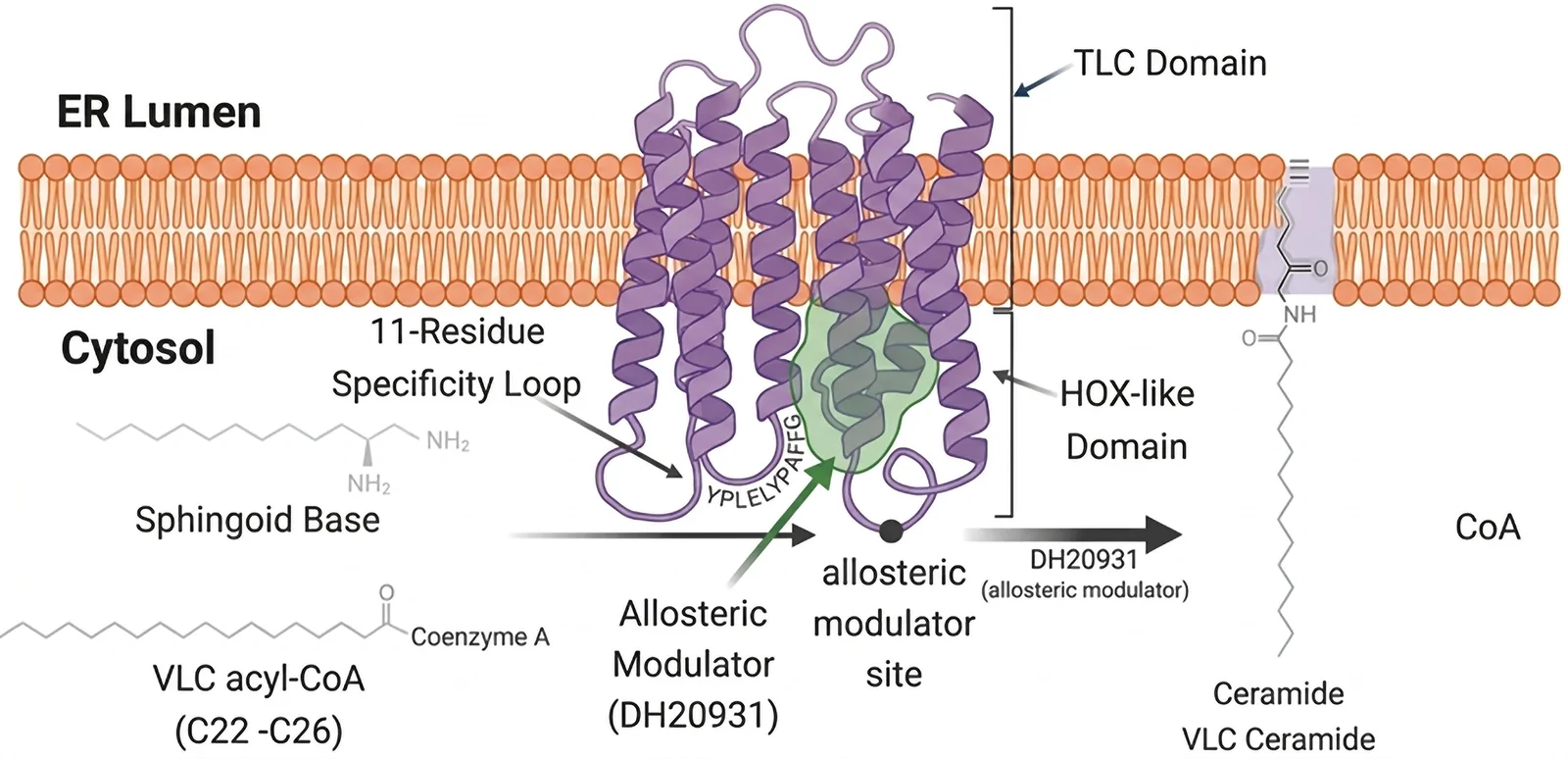
Molecular architecture and activation mechanism of human CerS2 The provided source for the three-dimensional structure of the human CerS2 protein is AlphaFold DB, with the specific UniProt accession number Q96G23. CerS2 is an integral ER membrane protein, possessing a luminal catalytic TLC domain and a cytosolic homeodomain-like (HOX-like) domain. A specific cytosolic 11-residue specificity loop, defined by the essential YPLELYPAFFG sequence shown, is crucial for substrate selection. The active site of the enzyme is on the cytosolic face, where it selectively utilizes a generic sphingoid base and VLC (C22-C26) acyl-CoAs (substrates shown bottom left). The diagram illustrates that the small molecule activator DH20931 binds to a distinct, cytosol-accessible allosteric pocket (green-shaded area), allosterically increasing the synthesis of VLC ceramides (structure shown right) while releasing CoA.

The activity of CerS2 is further governed by a complex network of post-translational modifications [39]. Phosphorylation at the C-terminal domain by Casein Kinase 2 has been shown to enhance enzymatic activity, while deacetylation by the mitochondrial protein Sirtuin 3 also increases its function and promotes ceramide accumulation within mitochondria [40,41]. Most notably, CerS2 is uniquely subjected to allosteric inhibition by sphingosine-1-phosphate [13,42]. Unlike other isoforms, CerS2 contains two motifs like those found in S1P receptors, allowing S1P to act as a non-competitive inhibitor [13]. This establishes a direct feedback loop between the opposing arms of the sphingolipid balance, where survival signals actively suppress the production of pro-apoptotic ceramides [13,43].

## Discovery of DH20931 and AI-accelerated pharmacology

The development of DH20931, a novel biisoquinoline derivative [33], represents a success of modern, artificial intelligence-accelerated drug discovery, in which the critical component was the use of AlphaFold to predict the three-dimensional structure of the CerS2 protein [32]. AlphaFold’s high-resolution structural models revealed novel binding pockets within the enzyme. DH20931 turned out to be a selective agonist of CerS2 with nanomolar potency. According to Swiss-ADME analysis, the molecule possesses favorable drug-like properties and adheres to the Lipinski Rule of Five [32,44–46]. DH20931 has a molecular weight of 358.4 Da, a lipophilicity (Log P) of 2.8, and demonstrates selectivity for cancer cells, which are often more dependent on altered sphingolipid metabolism than normal tissues [32]. The clinical development progress for DH20931 includes the completion of preclinical studies and a planned Investigational New Drug (IND) filing, with first-in-human Phase I clinical trials.

## Mechanisms of CerS2-induced lipotoxicity and ER stress

The activation of CerS2 and the subsequent accumulation of its VLC-ceramide products initiate a form of cellular stress known as lipotoxicity [47]. This process is not a passive metabolic side effect but an active program that disrupts organelle homeostasis, beginning with the physical alteration of the ER membrane and culminating in the activation of the terminal unfolded protein response (UPR) [47].

A growing body of evidence links the enzymatic activity of CerS2 to the induction of ER stress [32]. The incorporation of VLC-ceramides, which feature long and saturated acyl chains, into the ER membrane is hypothesized to decrease membrane fluidity and alter its biophysical properties [4,48–50]. These changes can directly impair the function of ER-resident proteins, such as chaperones and enzymes involved in protein folding [51–53]. In *CERS2* null models, the absence of these lipids leads to increased membrane fluidity and structural abnormalities in the ER, confirming their role in maintaining membrane architecture [36,54]. Since C24-ceramide forms interdigitated phases between the double bilayer [4], a decrease of VLC-ceramides by down-regulation of CerS2 may affect membrane properties by promoting vesicle adhesion, membrane fusion, and tubule formation [54].

Beyond general membrane disruption, elevated VLC-ceramides have a specific and direct impact on the ER-associated degradation (ERAD) machinery [55]. ERAD is the primary quality control system of the cell, which is responsible for identifying and removing misfolded proteins from the ER lumen [56–58]. VLC-ceramides physically restrict the function of this machinery by hindering the extraction of ubiquitinated protein substrates from the ER membrane [47,59,60]. This leads to a proteostatic crisis characterized by the rapid accumulation of misfolded proteins, which in turn triggers UPR [61].

The UPR signals through three primary sensors: protein kinase RNA (PKR)-like ER kinase (PERK), inositol-requiring enzyme 1 α (IRE1α), and activating transcription factor 6 (ATF6) [62–64]. While the initial goal of this response is adaptive, the persistent and severe nature of the lipotoxic stress induced by CerS2 activation shifts the signaling toward a pro-apoptotic program [32]. The PERK branch is the key driver of this transition. Upon activation, PERK phosphorylates the translation initiation factor eIF2α, which temporarily reduces global protein synthesis while paradoxically promoting the translation of activating transcription factor 4 (ATF4) [32]. ATF4 subsequently induces the expression of C/EBP homologous protein (CHOP), a master pro-apoptotic transcription factor [65–68]. Under conditions of irresolvable stress, sustained CHOP expression directs the death program by up-regulating factors such as p53 up-regulated modulator of apoptosis (PUMA), a member of the B-cell lymphoma 2 (Bcl-2) family that triggers mitochondrial outer membrane (MOM) permeabilization [69–72]. Building upon this mechanistic framework, recent studies demonstrate that DH20931 selectively intercepts this cascade at the level of CerS2 activation, lipotoxic ER stress, and PERK phosphorylation, thereby effectively shifting the UPR from an adaptive phase to an accelerated pro-apoptotic program via the ATF4/CHOP/PUMA axis [32] (Figure 2).

**Figure 2:**
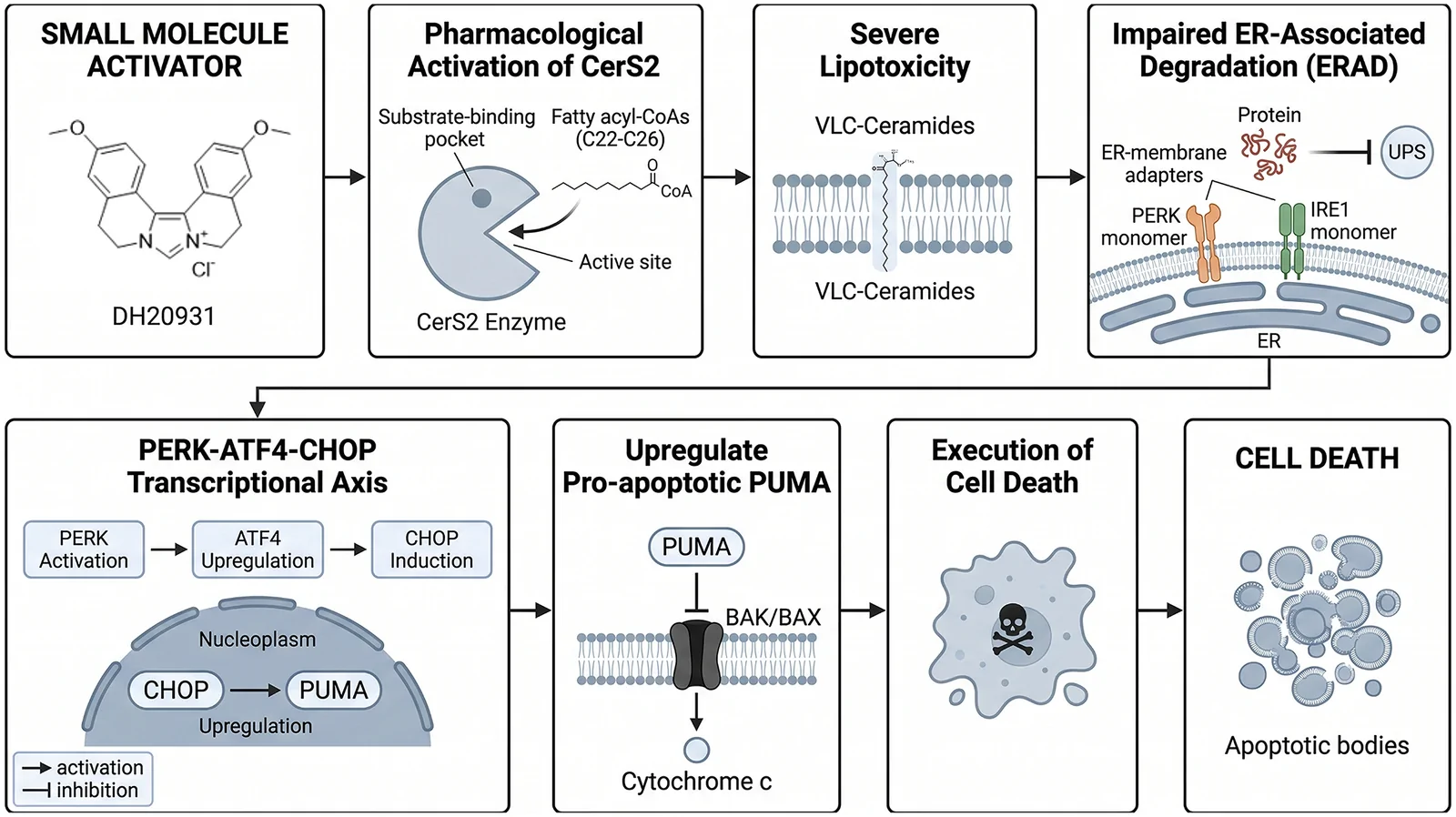
Mechanism I: Lipotoxic ER stress Pharmacological activation of CerS2 by DH20931 leads to an accumulation of VLC-ceramides, initiating severe lipotoxicity. This lipid imbalance physically impairs the ERAD machinery, causing a proteostatic crisis. The resulting chronic stress triggers the PERK-ATF4-CHOP transcriptional axis, which up-regulates the pro-apoptotic factor PUMA to execute cell death.

## The calcium signaling connection: CerS2-IP3R1 interaction at mitochondrial-associated membranes

While the UPR provides a transcriptional path to cell death, CerS2 activation simultaneously engages a more rapid executioner pathway centered on the dysregulation of intracellular Ca^2+^ signaling. This mechanism involves a critical dialogue between the ER and the mitochondria, the cell’s primary calcium reservoir and its metabolic powerhouse, respectively [73–76].

A recent 2026 report uncovers a novel mechanistic link: a direct physical interaction between CerS2 and the Inositol 1,4,5-trisphosphate receptor 1 (IP3R1) [32], which is a ligand-gated Ca^2+^ channel on the ER membrane [77,78]. Pharmacological activation of CerS2 with DH20931 promotes this interaction, facilitating the formation of an active complex that enhances the release of stored Ca^2+^ from the ER lumen [32]. This finding establishes CerS2 not just as a metabolic enzyme but as a signaling scaffold capable of directly modulating Ca^2+^ ion flux.

The consequences of this Ca^2+^ release are amplified by the structural connections between the ER and mitochondria at specialized contact sites known as mitochondria-associated membranes (MAMs) [79–81]. These MAMs create signaling microdomains where the local concentration of Ca^2+^ can reach extremely high levels, far exceeding the concentration in the bulk cytosol [79]. CerS2 activity appears to be an architectural modulator of these sites; treatment with DH20931 has been shown to reduce the physical distance between the ER and mitochondria [32], which is a critical parameter for efficient mitochondrial Ca^2+^ uptake [73,82,83]. Structurally, MAMs operate as lipid raft-like microdomains where sphingolipid and ceramide accumulation strictly regulates membrane architecture and inter-organelle contact [84,85]. By remodeling the lipid environment at these sites, VLC-ceramides likely induce structural packing that stabilizes the protein tethers connecting the two organelles, effectively tightening the gap for Ca^2+^ transfer and subsequent lipotoxicity [30,86].

This optimized transfer funnels Ca^2+^ directly into the mitochondrial matrix through the low-affinity mitochondrial calcium uniporter [87,88]. While a moderate influx of Ca^2+^ is beneficial for energy production, an excessive and sustained overload is a potent death signal [89–91]. Mitochondrial Ca^2+^ overload triggers the opening of the mitochondrial permeability transition pore (PTP), leading to the collapse of the mitochondrial membrane potential, a burst of reactive oxygen species, and the release of cytochrome c into the cytosol [92–95]. Once released, cytochrome c binds to Apaf-1, assembling the apoptosome and activating the caspase cascade [96–98] (Figure 3). Thus, the DH20931/CerS2-mediated sequential-mechanism model—combining a slow transcriptional death response (UPR) through the PERK-ATF4-CHOP axis with a fast signaling-based response (Ca^2+^ flux)—creates an important system to ensure cancer cell death [32].

**Figure 3:**
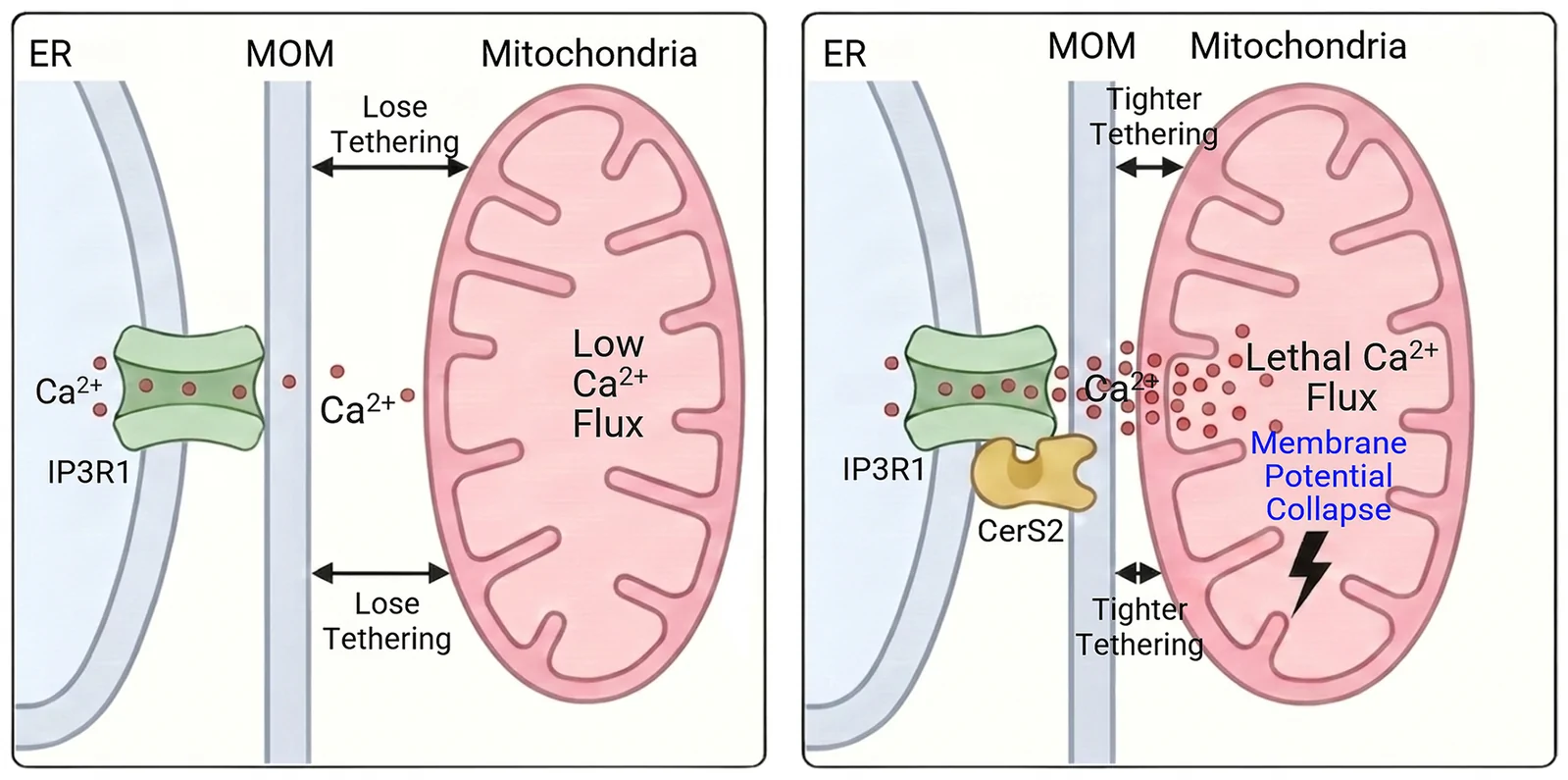
Mechanism II: DH20931 acts as a molecular glue to tighten the ER-mitochondria synapse (**A**) In the absence of CerS2 activation, the physical distance between the ER and the mitochondria results in loose tethering. Ca^2+^ released from the ER-resident IP3R1 channel dissipates into the cytosol, leading to low mitochondrial Ca^2+^ flux. (**B**) Treatment with the small molecule DH20931 activates CerS2, promoting its recruitment to MAMs. CerS2 serves as a physical bridge, interacting with the ER-resident IP3R1 and the MOM. This interaction facilitates tighter tethering, funneling a lethal influx of Ca^2+^ directly into the mitochondria. The resulting Ca^2+^ overload triggers the opening of the PTP, leading to mitochondrial membrane potential collapse and the subsequent release of cytochrome c, initiating the apoptotic cascade.

## CerS2 and IP3R1 interaction in thapsigargin-mediated ER-mitochondrial Ca^2+^ homeostasis

Recent studies have elucidated a novel regulatory axis thapsigargin (Tg) involving CerS2 and IP3R1 in the context of Ca^2+^ homeostasis. While Tg is traditionally recognized as a potent inhibitor of the sarco/endoplasmic reticulum-ATPase pump [99–101], recent findings suggest a more complex role in modulating inter-organelle communication [32]; Tg mimics the effects of DH20931 by inducing a physical and functional interaction between CerS2 and IP3R1. This interaction occurs at MAMs, where the physical distance between the endoplasmic reticulum and mitochondria is reduced to facilitate tighter organelle tethering [32]. Furthermore, it was shown that this treatment promotes the recruitment of CerS2 to the IP3R1 complex, effectively scaffolding a signaling microdomain. This coupling stabilizes the IP3R1 channel in a state that facilitates the rapid release of Ca^2+^ from the ER lumen directly into the mitochondrial matrix. The sudden influx of mitochondrial Ca^2+^ can trigger pro-apoptotic signaling pathways, a critical mechanism for overcoming chemoresistance in aggressive cancers [102–104].

The identification of this pathway provides a novel functional role for CerS2, positioning it as a key regulator of ER-mitochondria transfer. Modulating the Ca^2+^ flux may offer a promising strategic advantage in cancer cells. While the full clinical potential remains under investigation, the combination of Tg and DH20931 may represent a potent therapeutic strategy. By synergistically enhancing the CerS2–IP3R1 interaction, these agents could maximize mitochondrial overload to selectively induce cytotoxicity in cancer cells, including difficult-to-treat TNBC cells.

The clinical translation of Tg and its derivatives has faced a complex journey, primarily due to the compound’s potent but non-specific cytotoxicity [105]. While Tg is a gold-standard laboratory tool for inducing ER stress and Ca^2+^ signaling, its transition to the clinic has required innovative prodrug strategies to shield healthy tissues from its effects. The most advanced clinical derivative is mipsagargin, a Tg-based prodrug designed to be activated only by prostate-specific membrane antigen. This antigen is highly expressed in the vasculature of various solid tumors, such as hepatocellular carcinoma [106] and glioblastoma. While mipsagargin is reached phase II clinical trials (NCT02876003), the studies were concluded without moving phase III due to a strategic business decision by the sponsor rather than safety or efficacy issues. Further studies are required to determine the precise kinetics of this synergy and the long-term safety profile of such a combination, but these findings mark a significant step forward in understanding the non-canonical roles of ceramide synthases in oncogenic signaling.

## The CerS2 paradox: context-dependent roles in human malignancies

Despite the established pro-apoptotic potential of CerS2 activation, its role in oncology is characterized by a complex functional dichotomy. Widely recognized as tumor metastasis-suppressor gene 1, CerS2 overexpression has been shown to significantly impair the invasion and migration of various malignancies, including breast, prostate, bladder, hepatic, cervical, pheochromocytoma, and lung cancers [17–25,107–109]. A primary mechanism for this suppression, particularly in breast cancer, involves the inhibition of V-ATPase activity, which subsequently reduces the activation of matrix metalloproteinases and limits extracellular matrix remodeling [19]. While primarily functioning as a tumor suppressor, CerS2 can appear inert or even pro-tumorigenic in specific contexts [110,111]. Resolving this CerS2 paradox is essential for developing targeted interventions and requires a granular understanding of the enzyme’s net operational output, which is modulated by alternative splicing, endogenous inhibition, and metabolic shunting. These findings suggest that in many aggressive tumors, the loss of CerS2 function is a key step in cancer progression [107,108,112,113].

In the specific context of breast cancer, transcriptomic data indicates a significant elevation of *CERS2*, *CERS4*, and *CERS6* mRNA levels in malignant tumors compared with adjacent normal tissue [114]. This increase in mRNA during early-stage tumorigenesis may reflect a cellular stress response to the oncogenic environment. However, as the disease progresses—particularly in TNBC—this mRNA may be sequestered, improperly translated, or targeted for degradation. Multiple clinical datasets confirm a critical discrepancy; while mRNA levels may remain elevated, low CerS2 protein expression consistently serves as a poor prognostic factor, correlating with increased lymph node metastasis and reduced overall survival [108,112]. In non-small cell lung cancer, high mRNA expression of *CERS2* is a poor prognostic marker [115]. In prostate cancer, CerS2 has been shown to inhibit carcinogenesis by promoting the degradation of the oncoprotein β-catenin [116]. In bladder cancer, CerS2 regulates β-catenin signaling in a subset of stem-like cancer cells and sensitizes them to cisplatin treatment. In these studies, the mechanism of CerS2 was identified through dephosphorylation of β-catenin mediated by inactivation of PP2A and dissociation of PP2A-β-catenin [117]. Taken together, these findings suggest that in many aggressive tumors, the loss of CerS2 function is a key step in cancer progression. Thus, the aggressive tumors eventually silence the functional output of CerS2 to evade the apoptotic Ca^2+^ flux recently identified as a key CerS2-mediated executioner pathway [32] (Figure 4).

**Figure 4:**
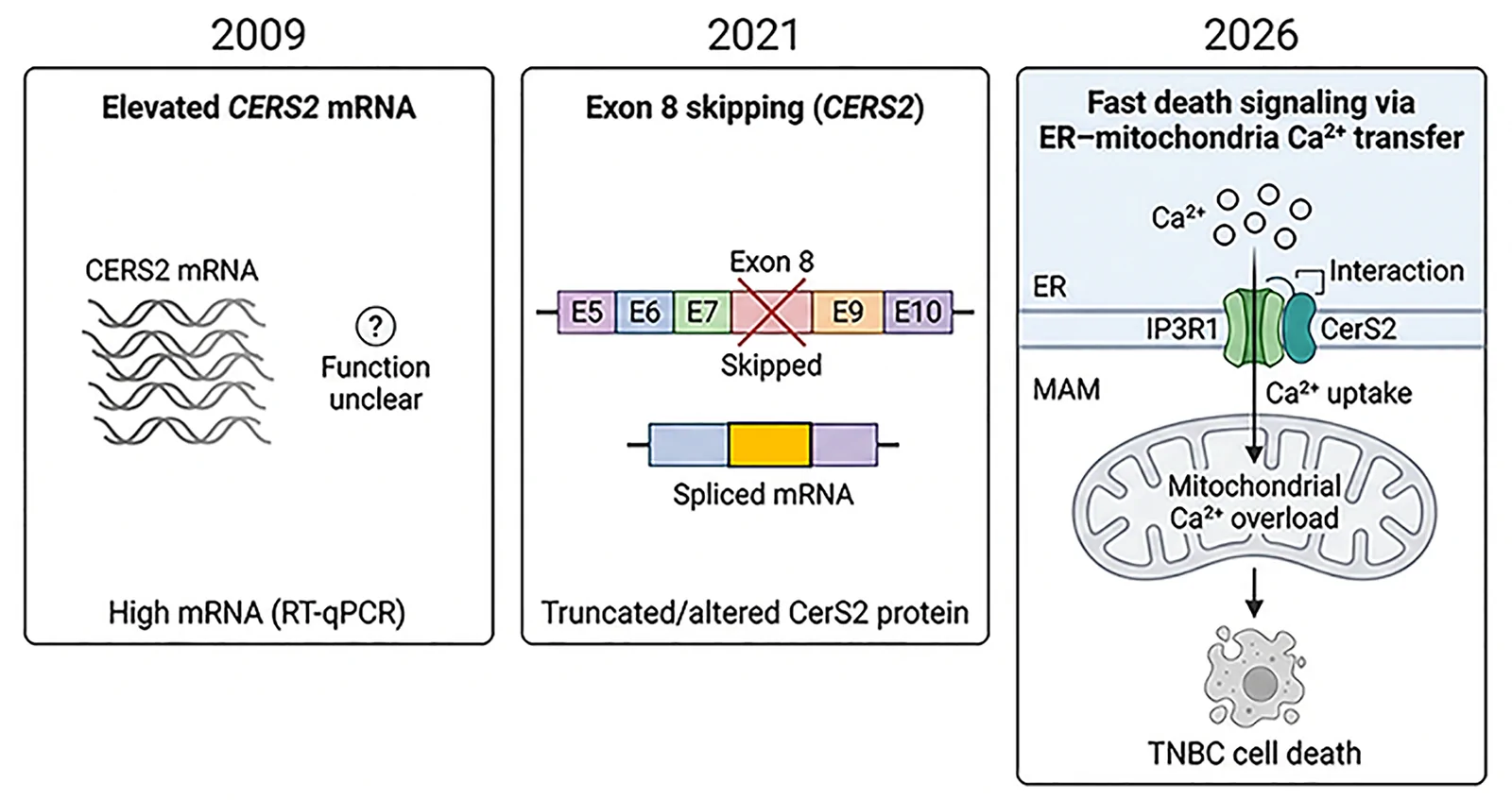
*CERS2* functional silencing model in aggressive TNBC This timeline illustrates the progression of research into the relationship between the *CERS2* gene and TNBC cell viability, culminating in a defined cell death signaling pathway. In 2009, studies identified elevated *CERS2* mRNA levels in aggressive breast cancer tissues (luminal B/TNBC) using RT-qPCR. Early hypotheses speculatively characterized *CERS2* as a potential tumor promoter based solely on transcript levels, though its exact functional output and mechanistic role remained unclear [114]. By 2021, the molecular mechanism was identified as exon 8 skipping within the *CERS2* gene, leading to a spliced mRNA and the production of a truncated or altered CerS2 protein. Consequently, this variant is unable to synthesize C22–26 ceramides and results in failed tethering at MAMs. This phenomenon underscores the functional silencing model, wherein high total *CERS2* mRNA counts mask a functional protein deficiency that allows aggressive TNBC cells to evade cell death signaling [18]. In 2026, the specific fast death signaling mechanism was characterized: the truncated CerS2 protein (teal) interacts with the IP3R1 calcium channel (green) within the MAM region of the ER. This interaction facilitates increased Ca^2+^ transfer from the ER, leading to enhanced Ca^2+^ uptake and subsequent mitochondrial Ca^2+^ overload. This overload triggers downstream signaling, ultimately resulting in TNBC cell death [32].

Another mechanism for silencing CerS2 involves direct protein inhibition. In glioblastoma, the anti-apoptotic protein Bcl2L13 is often overexpressed and binds directly to CerS2, blocking its enzymatic activity and promoting therapy resistance [118]. This suggests that in some cases, the cancer cell does not down-regulate the enzyme but instead deploys a molecular brake to prevent ceramide synthesis. Furthermore, in colorectal cancer, it is hypothesized that the pool of VLC-ceramides produced by CerS2 is rapidly diverted by the enzyme UDP-glucose ceramide glucosyltransferase into the synthesis of pro-proliferative glycosphingolipids. In this context, CerS2 acts as a supplier for a metabolic pipeline that drives tumor growth rather than death [111].

While resolving these varied, context-dependent mechanisms presents a compelling therapeutic avenue, the current body of evidence does not support the use of CerS2 agonists like DH20931 as universal, pan-cancer agents. Furthermore, proposing complex personalized medicine pipelines to identify susceptible tumors across all malignancies remains clinically unfeasible at present. Instead, the translational potential of DH20931 is best understood through its targeted efficacy in strictly controlled *in vitro* and *in vivo* models of specific, aggressive malignancies, most notably TNBC and colorectal cancer. In these defined contexts, DH20931 successfully exploits intrinsic sphingolipid metabolic vulnerabilities, triggering IP3R1-mediated mitochondrial calcium overload and subsequent lipotoxicity. By grounding our perspective in these concrete preclinical models rather than broad, pan-cancer applications, we highlight that the utility of DH20931 lies in targeting tumor types that are already proven to be selectively vulnerable to this distinct apoptotic axis.

## Preclinical efficacy of DH20931 and clinical outlook

The preclinical development of DH20931 offers a strategic solution to the functional silencing of CerS2 observed in aggressive malignancies. By acting as a potent agonist, DH20931 bypasses endogenous evasion mechanisms—such as isoform switching or proteasomal turnover—to restore the enzyme’s tumor-suppressive capacity. Its efficacy is primarily driven by a unique two-hit apoptotic mechanism that targets cancer cell homeostasis at the intersection of lipid metabolism and organelle signaling. The first hit involves the acute induction of lipotoxic ER stress through the rapid stimulation of VLC-ceramide synthesis. This metabolic shift triggers the pro-apoptotic ATF4-CHOP-PUMA signaling axis, priming the cell for execution (Figure 2). The second, and perhaps more definitive hit, involves structural reorganization at the MAMs. DH20931 promotes novel physical interaction between CerS2 and the ER-resident Ca^2+^ channel IP3R1. This interaction facilitates the tight tethering of the ER to the mitochondria, enabling a lethal flux into the mitochondrial matrix. This sudden Ca^2+^ overload leads to the loss of mitochondrial membrane potential and the subsequent release of cytochrome c, ensuring rapid and irreversible commitment to apoptosis (Figure 3). In orthotopic xenografts and patient-derived xenograft models of TNBC, DH20931 has demonstrated significant tumor growth inhibition with a favorable safety profile, showing no observable systemic toxicity or significant weight loss [32].

The clinical utility of DH20931 will be further amplified when integrated into combination therapy frameworks with standard-of-care FDA-approved chemotherapeutic agents, such as anthracyclines (e.g. doxorubicin) [119], taxanes (e.g. paclitaxel) [120,121], gemcitabine [122], and platinum-based compounds (e.g. carboplatin) [123]. By inducing a power surge of toxic lipids and calcium within the cell, DH20931 destabilizes the already fragile homeostasis of aggressive cancer cells, rendering them far more susceptible to the DNA-damaging effects of platinum agents or the mitotic disruption caused by taxanes. This multimodal approach effectively will lower the cytotoxic threshold for these traditional therapies, potentially allowing for dose-reduction strategies that could mitigate systemic side effects and improve the therapeutic window for patients with intractable disease.

Looking ahead, DH20931 is poised to redefine the therapeutic landscape by serving as a sophisticated architect of the tumor immune microenvironment. While its current pharmacological profile suggests that monotherapy may not inherently trigger a strong T-cell response, its future value lies in its distinct departure from traditional adjuvant agents through the induction of profound ER stress. However, the immunological and biological properties of CerS2 synthesized C24:1 ceramide (nervonoyl-ceramide) in cancer are increasingly recognized as distinct from those of shorter-chain species like C16:0. While ceramides are broadly considered pro-apoptotic and tumor-suppressive, C24:1 exhibits unique roles in modulating the tumor microenvironment (TME), suppressing metastasis, and regulating inflammatory signaling. In ovarian cancer, the CerS2-C24:1 axis acts as a functional barrier to metastasis. Elevated C24:1 level suppresses the formation of lamellipodia, the actin-rich protrusions necessary for cancer cell motility and invasion [112].

The immunological role of C24:1 ceramide is characterized by its ability to influence immune cell recruitment and barrier integrity. In the tumor vasculature, C24:1 ceramide supports endothelial barrier function by stabilizing inter-endothelial junctions [124]. This stabilization leads to the inhibition of ICAM-1 and VCAM-1 expression, which effectively reduces leukocyte diapedesis—the infiltration of immune cells from the blood into tissues [124]. Furthermore, while C16:0 ceramide is frequently associated with pro-inflammatory TLR4 activation and systemic meta-inflammation, C24:1 can exert anti-inflammatory or homeostatic effects. For instance, CerS2 deficiency—which leads to the depletion of C24:1 ceramide—has been shown to aggravate inflammation and increase neutrophil infiltration in models of experimental colitis, underscoring the role of C24:1 ceramide in preserving immunological and barrier stability [29,125].

This biological framework suggests that the pharmacological restoration or elevation of VLC-ceramides could strategically reshape the TME. The future application of DH20931 is expected to capitalize on this by inducing a localized stress response that triggers immunogenic cell death (ICD) [126]. By activating the terminal PERK-ATF4-CHOP axis and inducing severe lipotoxic ER stress [32], DH20931 may facilitate the translocation of calreticulin (CRT) to the cell surface. This exposure of CRT provides a critical eat-me signal to dendritic cells, an established hallmark of immunogenic stress [127,128].

Simultaneously, the proteostatic crisis initiated by CerS2 activation is predicted to drive the extracellular release of high mobility group box 1 and ATP, which act as potent damage-associated molecular patterns [126]. By bridging vascular stabilization and the induction of ICD, DH20931 offers a unique therapeutic avenue to transform cold tumors into immunologically hot environments, potentially synergizing with existing checkpoint inhibitors.

By orchestrating these molecular signatures, DH20931 will effectively heat up immunologically cold tumors, enhancing the recruitment and activation of T-cells within the previously silent tumor stroma. When integrated into broader, next-generation adjuvant strategies alongside immune-priming agents, DH20931 is expected to transform the metabolic and structural landscape at the MAM interface into a state of high immunogenicity. This shift will create a vital window of vulnerability that can be strategically exploited by immune checkpoint inhibitors (ICIs), ultimately offering a potent, multi-modal pathway for overcoming resistance in refractory TNBC.

## Conclusion: the path forward for CerS2-targeted therapies

CerS2 has emerged as a master regulator of cell fate, positioned at a critical intersection of lipid metabolism, organelle signaling, and apoptosis. The synthesis of VLC-ceramides by this enzyme serves as a decisive molecular switch, capable of initiating a definitive cell death program through the simultaneous disruption of proteostasis and the mobilization of lethal mitochondrial Ca^2+^ flux. The recent discovery of DH20931 and its novel mechanism of promoting CerS2-IP3R1 interactions at the MAMs represents a major advancement in our ability to biophysically manipulate these inter-organellar contact sites for therapeutic benefit.

Looking forward, the inherent complexity of CerS2 regulation—characterized by alternative splicing, post-translational modifications, and varying metabolic landscapes—dictates that the next phase of development must focus on defining these context-specific vulnerabilities in rigorous preclinical models. A pivotal challenge for the field will be the development of vigorous companion diagnostics to accurately stratify patients. Such platforms must be capable of mapping the CerS2 landscape of a tumor, including the identification of non-functional splice variants (e.g. exon 8 skipping) and the quantification of endogenous inhibitors like Bcl2L13, which may otherwise impede therapeutic activation.

As DH20931 advances toward clinical trials, it heralds the arrival of a new class of smart metabolic activators designed to exploit the fundamental structural and biochemical vulnerabilities of cancer cells while preserving the integrity of normal tissue. Future research must now delve deeper into the biophysical properties of VLC-ceramides and their influence on membrane curvature and protein scaffolding at the MAM interface. Furthermore, exploring how CerS2 activation can be integrated with emerging modalities—such as metabolic reprogramming agents or ICIs—will be essential to overcoming adaptive resistance. Ultimately, refining our understanding of this proteostatic-mitochondrial axis will provide a transformative framework for improving clinical outcomes in patients with aggressive, refractory malignancies that have historically evaded standard-of-care therapies.

## Open Access

Open access for this article was enabled by the participation of University of Florida in an all-inclusive Read & Publish agreement with Portland Press and the Biochemical Society.

## Abbreviations

ATF 4: activating transcription factor 4
ATF 6: activating transcription factor 6
Bcl2: B-cell lymphoma 2
Ca^2+^: calcium
CerS2: ceramide synthase 2
CHOP: C/EBP homologous protein
CRT: calreticulin
ER: endoplasmic reticulum
ERAD: ER-associated degradation
ICD: immunogenic cell death
ICI: immune checkpoint inhibitors
IP3R1: inositol 1,4,5-trisphosphate receptor 1
IRE1α: inositol-requiring enzyme 1 α
MAM: mitochondria-associated membrane
MOM: mitochondrial outer membrane
PERK: protein kinase RNA (PKR)-like ER kinase
PTP: permeability transition pore
PUMA: p53 upregulated modulator of apoptosis
Tg: Thapsigargin
TLC: TRAM-Lag1-CLN8
TME: tumor microenvironment
TNBC: triple-negative breast cancer
UPR: unfolded protein response
VLC: very-long-chain
